# Implementation and evaluation of a harm-reduction model for clinical care of substance using pregnant women

**DOI:** 10.1186/1477-7517-9-5

**Published:** 2012-01-19

**Authors:** Tricia E Wright, Renee Schuetter, Eric Fombonne, Jessica Stephenson, William F Haning

**Affiliations:** 1 Department of Obstetrics, Gynecology and Women's Health, University of Hawaii John A. Burns School of Medicine, 1319 Punahou St., Ste. 824, Honolulu, HI 96826 USA; 2 Perinatal Addiction Treatment of Hawaii, 845 22nd. Ave., Honolulu, HI 96816. USA; 3 Department of Child Psychiatry, McGill University, 845 Sherbrooke Street West, Montreal, Quebec, H3A 2T5, Canada; 4 Department of Psychiatry, University of Hawaii John A. Burns School of Medicine, 1356 Lusitana St., 4th Floor, Honolulu, HI 96813 USA

**Keywords:** Methamphetamines, Harm Reduction, Pregnancy, Birth Outcomes, Tobacco

## Abstract

**Background:**

Methamphetamine (MA) use during pregnancy is associated with many pregnancy complications, including preterm birth, small for gestational age, preeclampsia, and abruption. Hawaii has lead the nation in MA use for many years, yet prior to 2007, did not have a comprehensive plan to care for pregnant substance-using women. In 2006, the Hawaii State Legislature funded a pilot perinatal addiction clinic. The Perinatal Addiction Treatment Clinic of Hawaii was built on a harm-reduction model, encompassing perinatal care, transportation, child-care, social services, family planning, motivational incentives, and addiction medicine. We present the implementation model and results from our first one hundred three infants (103) seen over 3 years of operation of the program.

**Methods:**

Referrals came from community health centers, hospitals, addiction treatment facilities, private physician offices, homeless outreach services and self-referral through word-of-mouth and bus ads. Data to describe sample characteristics and outcome was obtained prospectively and retrospectively from chart abstraction and delivery data. Drug use data was obtained from the women's self-report and random urine toxicology during the pregnancy, as well as urine toxicology at the time of birth on mothers, and urine and meconium toxicology on the infants. Post-partum depression was measured in mothers with the Edinburgh Post-Partum depression scale. Data from Path clinic patients were compared with a representative cohort of women delivering at Kapiolani Medical Center for Women and Children during the same time frame, who were enrolled in another study of pregnancy outcomes. Ethical approval for this study was obtained through the University of Hawaii Committee for Human Studies.

**Results:**

Between April 2007 and August 2010, 213 women with a past or present history of addiction were seen, 132 were pregnant and 97 delivered during that time. 103 live-born infants were delivered. There were 3 first-trimester Spontaneous Abortions, two 28-week intrauterine fetal deaths, and two sets of twins and 4 repeat pregnancies. Over 50% of the women had lost custody of previous children due to substance use. The majority of women who delivered used methamphetamine (86%), either in the year before pregnancy or during pregnancy. Other drugs include marijuana (59.8%), cocaine (33%), opiates (9.6%), and alcohol (15.2%). Of the women served, 85% smoked cigarettes upon enrollment. Of the 97 women delivered during this period, all but 4 (96%) had negative urine toxicology at the time of delivery. Of the 103 infants, 13 (12.6%) were born preterm, equal to the state and national average, despite having many risk factors for prematurity, including poverty, poor diet, smoking and polysubstance use. Overwhelmingly, the women are parenting their children, > 90% retained custody at 8 weeks. Long-term follow-up showed that women who maintained custody chose long-acting contraceptive methods; while those who lost custody had a very high (> 50%) repeat pregnancy rate at 9 months post delivery.

**Conclusion:**

Methamphetamine use during pregnancy doesn't exist is isolation. It is often combined with a multitude of other adverse circumstances, including poverty, interpersonal violence, psychiatric comorbidity, polysubstance use, nutritional deficiencies, inadequate health care and stressful life experiences. A comprehensive harm reduction model of perinatal care, which aims to ameliorate some of these difficulties for substance-using women without mandating abstinence, provides exceptional birth outcomes and can be implemented with limited resources.

## Background

Methamphetamine (MA) use by pregnant women has grown into the one of the greatest public health challenges of the 21^st ^century. Hawai'i arguably leads the nation in the use of MA, especially among women, given the highest percentages of female arrestees testing positive for MA use are located in Honolulu (50%), San Jose (42.8%), Phoenix (41.7%), Salt Lake City (37.7%), and San Diego (36.8%) [1]. In addition, the use of MA by women of childbearing age is increasing [2-4]. Indeed MA has become the primary substance compelling drug treatment during pregnancy [5]. Yet despite this epidemic, prior to 2007, Hawaii did not have a comprehensive plan to care for pregnant substance-using women. In this paper, we review what is known about MA use in women and MA in pregnancy; the implementation model for a perinatal clinic; and results from the first 97 women to deliver.

### Women and Methamphetamine

Men's rates of substance use disorders are nearly twice as high as the rate for females (11.9 vs. 6.1 percent) [6]. However, women use MA at similar rates as do men. The NSDUH 2009 data [6] showed that 0.2% of both men and women used MA in the past year. In 2007, females accounted for 40% of stimulant-related ED visits and 45% of MA-related treatment admissions in the United States [7]. In comparison, men are three times more likely to abuse alcohol and twice as likely to use cocaine than women [6]. Reasons for this discrepancy are multiple and need to be addressed during treatment.

### Co-occurring Disorders

Women are more likely to have co-occurring mental health disorders; as many as two thirds of women with substance use disorders may have a co-occurring mental health problem, including depression, post-traumatic stress disorder, panic disorder and/or eating disorder [8]. They are more likely to be victims of domestic violence, incest, rape, sexual assault, and child physical abuse. This victimization has been linked to many negative outcomes, including posttraumatic stress disorder (PTSD), depression, anxiety, suicidal behavior and low self-esteem among women in the general population [9]. Women who are survivors of childhood sexual assault are more likely to use alcohol and other drugs [10,11]. Posttraumatic stress disorder is strongly associated with stimulant use disorders, including MA [12].

Methamphetamine, increasing the synaptic levels of not only dopamine, but also norepinephrine and serotonin, is a powerful antidepressant at first, which may partially explain its allure to women. Semple et al [13] showed that women who use MA had significantly higher Beck Depression Index scores than men who used MA. MA users in general have high levels of psychiatric symptoms and psychological problems. One study showed that women scored higher than men on every psychological symptom at treatment entry, especially anxiety, depression, interpersonal sensitivity, obsessive-compulsive disorder and somatization [14].

### Weight Loss

In addition, many women begin using MA in order to lose weight, as many as 36% of women in one study vs. 7% of men [15]. Piran and Robinson [16] showed high rates of stimulant use among women with eating disorders. Corstorphine et al [17] showed that women with eating disorders who were victims of childhood sexual assault were more likely to use alcohol, cocaine or amphetamines. Gender roles stressing the thin-ideal body image may exacerbate the problem, especially among white, Native American and Pacific Island women [18].

### Caregiver role

Women who must hold down multiple jobs, including caring for children often turn to MA to give them the extra energy they need to be "supermom." Though men more often than women use methamphetamine to "work more," and are more likely to be employed than women, women were more likely to use MA to stay awake [15].

### Cost

The cost of drugs may play a large role in initiation. Women are more likely to live in poverty, and when they do get drugs, it is usually from a partner who is also using [15]. Methamphetamine acts longer than cocaine and other stimulants and before precursor legislation, cost less. One nickname for the drug is "poor-man's cocaine." In addition, many studies have shown that women MA users are more likely to be homeless [19].

### Methamphetamines and Pregnancy

Despite the widespread nature of MA use in women of childbearing age, little research has been done on its use during pregnancy. Most prior drug use research was conducted in response to the crack cocaine epidemic of the early 1980s. Much remains unknown about the effects of *in utero *MA exposure and fetal and infant development. No consistent teratological effects of *in utero *MA exposure on the developing human fetus have been identified [20,21]. Given that women with substance use disorders suffer from chaotic lifestyles, research on drug use during pregnancy is fraught with difficulties. Studies of MA-exposed infants suffer from methodological problems such as poor compliance, small sample size and multiple other confounding variables, such as the effects of poverty, poor diet, and tobacco use, which in studies of other drug use during pregnancy have been found to be as harmful if not more so than the drug use itself [22] Learning from past mistakes from the crack cocaine epidemic, Barry Lester and colleagues initiated the Infant Development, Environment, and Lifestyle (IDEAL) study in 2002 to study the effects of prenatal methamphetamine exposure. Investigators have enrolled 1,618 mothers at four clinical centers in cities where methamphetamine use is problematic, including Honolulu. Thus far the results from the IDEAL study indicate that methamphetamine is associated with fetal growth restriction [23], but no measurable difference in psychological functioning by age three [24].

There is some limited data on the effects of MA use on maternal complications during pregnancy [25-30]. With the exception of Cox et al [29], the numbers in these studies are all small and suffering from the methodological problems noted above. The Cox study showed increased risks of hypertension complicating pregnancy, premature rupture of membranes, placenta previa, placental abruption, premature delivery, precipitate labor, infection of amniotic cavity, intrauterine death, and poor fetal growth when compared with non-substance using women, but when compared with cocaine-using women, these risks were all less, with the exception of hypertension complicating pregnancy, which was increased over cocaine.

Studies looking at birth weight and gestational age show conflicting results, with most showing slightly smaller babies and lower gestational ages [26,27,31,32]. Studies on neurodevelopment [33-37] have shown MRI differences and small differences in performances on some developmental measures, but with the exception of the IDEAL study, longitudinal data is missing.

### Harm Reduction and Pregnancy

With the exception of methadone maintenance, which is currently the standard of care for opiate-dependent pregnant women, harm reduction programs for pregnant substance-using mothers are rare, especially in the United States. As stated by McGourty and Chasnoff: "From a public health perspective, the concept of harm reduction does not have a role in the care of pregnant women [38]." Part of the problem is in the definition of harm reduction. It refers to both a philosophical approach and specific types of programs or interventions [39]. Perhaps McGourty and Chasnoff misconstrued that harm reduction means simply tolerating drug or alcohol use during pregnancy, which, especially in the case of alcohol, goes against the necessary teaching that no amount of alcohol is known to be safe during pregnancy [40].

Prenatal care has been shown to ameliorate many of the complications of drug use during pregnancy [41], so it makes empiric sense that a harm reduction philosophy as it facilitates entry into prenatal care, would decrease pregnancy complications. There has been little written on harm reduction programs for pregnant women, mostly originating from the U.K. and Canada, though programs certainly exist in the U.S. The literature on harm reduction in pregnancy from the U.S. mainly focuses on harm reduction by reducing use, not specific programs. One specific program in Canada has shown great promise with a harm reduction approach.

Poole stated in her evaluation of the Sheway project in British Columbia [42]:

Particularly promising is harm reduction programming offered to high-risk, marginalized pregnant women and their support networks, an approach that has been shown to prevent FAS and other alcohol- and drug-related disabilities, support retention of custody, and increase the health and social stability of both mothers and children

Key components of a harm reduction program should focus on improving nutrition, decreasing smoking, decreasing alcohol and drug use, encouraging breastfeeding, promoting dental health and encouraging physical activity, encouraging early and continuing prenatal care and promoting social and community support [43].

This review of the literature has shown many of the important concepts that must be addressed when providing care to pregnant women with substance use disorders. There are a few dozen programs that specifically provide care for pregnant substance-using women, but little in the literature evaluating their effectiveness. These concepts were used to build a comprehensive program, which is described below.

## Methods

### Context

The clinic concept was developed starting in March 2005 when a group of community leaders started meeting. Funding proposals were developed through a collaboration with the University of Hawaii, the Hawaii Departments of Human Services and Health, ACOG Hawaii Section and the Salvation Army Family Treatment Center, led by one of the authors (TEW). When federal grant proposals were not funded, the decision was made to appeal to the Hawaii State Legislature for funding. The Hawaii State Legislature passed legislation establishing the clinic in 2006 with the initial sum of $400,000. The clinic model was developed with input from the University of Hawaii Ob/Gyn, Pediatrics and Addiction Medicine, and based on the successful Milagro clinic in Albuquerque, New Mexico. The clinic opened in April 2007 in a 1500 square foot house on the grounds of the Salvation Army Family Treatment Center.

### Clinic Mission

Our mission is to provide comprehensive perinatal clinical and social services to women with a history of substance abuse, in a warm home-like setting free of judgment, and supportive of each woman's unique path from pregnancy to capable parent.

### Program Goals

1. Improve birth outcomes in women who use drugs and/or alcohol during pregnancy.

2. Improve developmental and behavioral outcomes for children whose mothers used alcohol or other drugs during pregnancy.

3. Provide an educational environment for residents, students, and nurses to learn about the care of this high-risk population in a sensitive manner.

4. Provide educational resources to the community in order to decrease the prevalence of drug use among pregnant and postpartum women.

5. Provide an environment to promote research on substance abuse treatment, its effects on the mother, fetus, and child, and prevention.

### Clinic Services

The initial clinic services began with prenatal and postpartum care. Deliveries were done by residents and staff of the University of Hawaii at Kapiolani Medical Center for Women and Children and Queens Medical Center. Childcare was provided on site, transportation was provided either by taxi or by bus passes. Addiction psychiatry services were provided by addiction psychiatry and addiction medicine resident fellows under the supervision of one of the authors (WFH). Social services were provided on site, including case management, assistance with housing, substance abuse assessments and referral to more intensive treatment if indicated and desired. Group classes were provided including childbirth, parenting, smoking cessation, healthy eating, and relapse prevention. In addition, groups were established including self-nurturing group and craft groups to build self-esteem and enhance maternal infant bonding as well as relationship groups to address the needs of the women and their partners. Healthy food was provided to women while attending classes and clinic appointments, and by year three, we had obtained a grant from the Office of Hawaiian Affairs to provide a healing garden on site. With this garden, we were able to grow a good proportion of the fruits and vegetables provided to the women. While we were unable to engage the services of a dedicated pediatrician to meet goal (2) in the first three years of operation, collaboration and referrals were made with staff pediatricians at Kapiolani Medical center and sympathetic community pediatricians.

### Medical Care

Prenatal visits were scheduled every 2 weeks, given the high-risk nature of these pregnancies, and the ability to engage women more frequently in social services if they came for medical care. Ultrasounds were generally scheduled every 4-8 weeks after 20 weeks to watch for intrauterine growth restriction and promote maternal bonding. As much as possible, all laboratory tests were done on site. Each woman received testing and counseling for HIV and Hepatitis C. Addiction psychiatry and group classes were scheduled around clinic visits.

### Counseling approach

All clinic staff, including medical assistants and residents, was trained in Motivational Interviewing techniques [44], using a Stages of Change Model [45]. In addition, counseling was trauma informed [46,47], given high rates of childhood sexual assault in the clinic population. As much as possible, a patient-centered harm reduction approach was used. Women were encouraged to participate in as many or as few activities as they were comfortable with.

### Motivational Incentives

Contingency management, in which patients receive incentives or rewards for meeting specific behavioral goals (e.g., verified abstinence), has particularly strong, consistent, and robust empirical support across a range of types of drug use. Contingency management approaches are based on principles of behavioral psychology and operant conditioning, in which behavior that is followed by positive consequences is more likely to be repeated [48]. We received a grant from the local March of Dimes office to provide motivational incentives for women to help overcome barriers to clinic participation. Recognizing that getting clients through the door was the biggest barrier, the women received an initial $50 gift card for their first visit. This was reduced to $20 after the first year based on studies done by Petry and others recommending nominal and intermittent rewards [49]. After completion of the psychosocial assessment, they were awarded with an additional $20 gift card. In addition, they received picks from the "fishbowl" for each subsequent doctor's visit, to reward group attendance and taking steps toward goal achievement. The fishbowl had small tokens saying "good job," "small," or "large." Small gifts were usually baby or toiletry items of nominal cost and the large gifts were $50 gift cards.

### Ethical Approval

The University of Hawaii Committee on Human Studies reviewed the project and found it to be exempt from consent requirements in order to report clinical outcomes.

### Outcomes

Data to describe sample characteristics and outcome was obtained prospectively and retrospectively from chart abstraction and delivery data. Drug use data was obtained from the women's self-report and random urine toxicology during the pregnancy, as well as urine toxicology at the time of birth on mothers, and urine and meconium toxicology on the infants. Post-partum depression was measured in mothers with the Edinburgh Post-Partum depression scale, where a score of 10 or more is indicative of high levels of depressive symptomatology. For comparison, data on birth outcomes of Path clinic patients were compared with those of a representative cohort of women delivering at Kapiolani Medical Center for Women and Children during the same time frame, who had been recruited for the Pacific Research Center on Early Human Development study (PRCEHD). The data on this cohort has been presented in a previous paper [30].

### Statistical Analysis

Data was analyzed using SAS 9.2 (Cary, NC). Conventional descriptive statistics were used. Dichotomous data were compared using chi-square and continuous data compared using student T-test. In order to further evaluate program effectiveness, we developed an outcome score in 4 separate domains: birth outcomes, drug use, custody, and repeat pregnancy. For each domain, several indicators were considered and scored with a binary coding scheme where 1 indicated a poor outcome and 0 an outcome within the normal range (see table five footnotes for the list of indicators mapping each outcome domain). The indicators scores were then summed up into a total domain score that was subsequently dichotomized into a 0/1 binary summary score. For each domain score, we conducted a series of bivariate analyses of several candidate predictors, including engagement in prenatal care, parity, maternal medical condition, psychosocial variables (domestic violence, custody loss,..), drug use variables, and residential treatment status. Each candidate predictor was retained for logistic regression modeling based on a p-value < 0.25 in bivariate analysis. Multiple logistic regression models were subsequently performed with stepwise selection of candidate predictors. Throughout, a p-value of 0.05 was retained as the criterion for statistical significance. The fit of multiple logistic regression models was assessed with the Hosmer-Lemeshow statistic.

Between April 2007 and August 31, 2010, 213 women were seen for medical care. These women all had a history of addiction and were either actively using or in early abstinence and thus at risk for relapse. There were 132 pregnant mothers and 97 of those delivered 103 live-born infants during that time, including two sets of twins and 4 repeat pregnancies. There were 3 first-trimester spontaneous abortions and two 28-week interuterine deaths (IUFD) thought to be secondary to cytomegalovirus (CMV) exposure. Patients were seen for a mean of 7 prenatal visits (range 1-19). Demographics are shown in table 1.

**Table 1:** Demographics and clinical characteristics of Path women compared with a cohort of women delivering at the same hospital over the same timeframe

	*Path Clinic*		*Kapiolani*		*p-value*	
	Mean	SD	Mean	SD		
Age	28.3	6.5	28.6	6.6	0.65	
Gravidity	4.5	3.1	2.9	2.1	< 0.001	
Parity	2.3	2.2	1.2	1.4	< 0.001	
Abortions	1.2	1.9	0.7	1.1	0.01	
Gestational Age	38.5	2.4	38.5	2.0	0.83	
Birth Weight	3159.2	622.4	3189.9	552.2	0.61	
	N = 97	Percent	N = 872	Percent		
Native Hawaiian	47	49.5%	193	22.1	< 0.001	
Smoking at delivery	65	63%	87	10%	< 0.001	
Methamphetamines						
during pregnancy	68	66	22	2.5	< 0.001	

### Medical Conditions

There was a very high rate of asthma in the Path clinic group (28%) compared with the Kapiolani cohort (KC) (3.7%) p < 0.001. Chronic hypertension rates were also higher than in the KC sample (9.9% vs. 4.5% p = 0.02). In the Path sample, there were no cases of HIV and 6.2% had Hepatitis C. There were two cases of maternal heart disease, which were possibly related to MA use. One woman had pregestational diabetes. Co-occurring mental health disorders were present in 36% of the women, including 27% with depression, 10% with diagnosed PTSD, and 5% with bipolar disorder.

### Psychosocial Stressors

In the Path sample, there were high rates of reported interpersonal violence, including 55% with a history of child-abuse or neglect and 62.5% with a history of domestic violence. Ninety five percent of the women were on Medicaid.

### Drug use

MA was overwhelmingly the drug of choice for these women (86%), though twenty-four percent used more than one drug. Almost all women stopped or decreased the use of drugs, with equal amounts of women becoming abstinent in each trimester and only 6.3% continuing to use until delivery. Only 5% of the infants' toxicology screens were positive. Of the eight opiate-dependent women (one woman only admitted to using occasional "pills" including oxycodone), 5 were on methadone maintenance, 1 was on buprenorphine, and 2 women were detoxified before being admitted to residential treatment and before coming to the clinic. Drug use characteristics are shown in table 2.

**Table 2:** Substance Use Characteristics

*Drug Use*	*N = 97*	%
Methamphetamines	87	86.1
1st trimester	19	19.8
2nd trimester	22	22.9
3^rd ^trimester	21	21.9
Continuous	6	6.3
Cocaine	30	33
Marijuana	55	59.8
Opiates	9	9.6
Smoker	79	84
Alcohol	14	15.2 11-1st trimester use only
Baby toxicology + (mec or urine)	4	5.1
Smoking		
		
Non-Smoker	15	16
Quit during pregnancy	14	15
Decreased during pregnancy	34	36
Stayed same	30	32
Increased	1	1.1

Eighty four percent of the women smoked at the beginning of pregnancy, and only 14 percent of women were able to quit during pregnancy, though 34 percent decreased usage.

Pregnancy complications and birth outcomes are listed in table 3. There were higher rates of preeclampsia in the Path sample compared with the KC sample, but most other pregnancy complications, notably preterm delivery, low birth weight, and cesarean delivery rates showed no difference. We have no comparison data for infant outcomes in the KC data, but rates of newborn intensive care admissions in the Path sample are relatively low, and 88.1 percent of infants were discharged with their mothers. There were no cases of intraventricular hemorrhages, necrotizing enterocolitis or neonatal death. Of 58 neonates for whom we have full pediatric information, there was only one major anomaly diagnosed shortly after birth and 5 minor anomalies, including 3 hydroceles and 2 minor digit defects.

**Table 3:** Pregnancy complications

	*Path Clinic*		*Kapiolani*		*p-value*
	N	Percent	N	Percent	
IUGR	5	5.0%	N/A		
Preeclampsia	11	11.0	35	4.0	0.0025
Preterm labor	11	10.9	112	12.8	0.58
PPROM	6	5.9	28	3.2	0.16
Abruption	2	2.0	N/A		
Diabetes	8	8.0	113	12.9	0.22
Oligohydramnios	3	3.0	23	2.6	0.84
Previa	1	1.0	N/A		
Chorioamnionitis	6	5.9	N/A		
Non-reassuring fetal heart			N/A		
tones	6	5.9			
Polyhydramnios	6	5.9	N/A		
Cesarean section	34	33.0	252	29.0	0.39
Delivery < 37 weeks	13	12.6	131	15.0	0.70
Delivery < 32 weeks	1*	0.9	14	1.6	0.80
LBW	14	13.6	85	9.7	0.22
VLBW	2*	1.9	10	1.1	0.49
NICU admission	15	16.1	N/A		
Hypoglycemia	3	4.7	N/A		
Hyperbilirubin	11	10.8	N/A		
sepsis	1	1	N/A		
RDS	5	4.9	N/A		

* 1 set of twins. 1 very preterm birth, but 2 very low-birth weight infants.

EBL = estimated blood loss, LBW = low birth weight < 2500 g, VLBW = very low birth weight < 1500 g, NICU = neonatal intensive care unit, RDS = respiratory distress syndrome, IVH = interventricular hemorrhage

### Post-Partum

Data on post-partum follow-up, including incidence of post-partum depression, contraceptive methods, and repeat pregnancy rates are shown in table 4.

**Table 4:** Postpartum follow-up

	*n*	%
Postpartum Depression	22	21.4
LARC	28	28
Sterilization	14	14
OCPS/Ring	12	12
DMPA	7	7
None	15	15
Lost	22	22
Female Partner only	2	2
Repeat pregnancy	13	17

DMPA=depo medroxyprogesterone acetate, LARC=long-acting reversible contraception. OCPS=oral contraceptive pills.

Twenty-one percent of women screened positive for possible postpartum depression with an Edinburgh postpartum depression scale of over 10. Twenty eight percent of women chose long-acting reversible contraception and 14% chose sterilization. Overall, 63% of women chose some form of contraception immediately post-partum.

#### Analysis of poor outcomes

### Outcome analyses

Outcome analysis is presented in table 5. The table summarizes 4 independent logistic regression models for each of the 4 outcomes considered. For each outcome, only predictors that were significant at p < 0.05 are presented in the Table, with their corresponding odds-ratio adjusted for the effects of other predictors.

**Table 5:** Predictors of poor outcomes in 4 domains

Infant Poor Outcomes*	AOR	95% confidence interval
Parity	0.6	(0.4-0.9)
History of Domestic		
Violence	5.7	(1.3-26.6)
Maternal Medical		
Condition	4.6	(1.1-18.9)
		
Drug Use Poor Outcomes**		
Residential Treatement	0.17	(0.031-0.95)
Poor prenatal care	12.7	(2.8-57.1)
		
Custody Loss Poor Outcomes***		
Residential Treatement	0.035	(0.004-0.3)
3rd trimester drug use	7.9	(2.2-27.6)
		
Family Planning Poor Outcome****		
Custody loss	2.7	(1.02-6.9)

Note: each domain summarizes outcome as measured by the following indicator variables:

*preterm delivery, LBW, NICU admission, baby Length of stay > 4 days

**positive toxicology at birth (mom or baby), relapse within 6 months

***not discharged with baby (secondary to child welfare) or lost custody within 6 months.

****not on birth control postpartum or repeat pregnancy within 6 months

Infant outcomes were influenced by maternal parity (protective), maternal medical conditions, and domestic violence, when controlled for amount of prenatal care and homelessness. Drug use was most heavily influenced by residential treatment (highly protective) and lack of prenatal care. Loss of custody was also strongly associated with the protective presence of residential treatment (AOR = 0.035) and an eight-fold increase in risk due to third trimester drug use. Family planning failure was associated with custody loss.

## Discussion

This paper shows the success and challenges in caring for this high-risk population. Women in this study had high rates of co-occurring medical and mental health problems, high poverty rates, high rates of interpersonal violence, and high smoking rates. Despite these challenges, with the appropriate interventions and care, women with addictions can have relatively normal birth outcomes. Previous work by one of the authors showed a 4-fold increased risk of preterm delivery with methamphetamine use in this population [30]. We definitely did not see this increase, as our preterm delivery rate was not different from the cohort from the same hospital, as well as state and national averages (12.8 and 12.3 respectively) [50]. We did see increased preeclampsia rates, which are not surprising as asthma, chronic hypertension, and methamphetamine use are all associated with preeclampsia [29].

By providing a safe environment to obtain prenatal care, we ameliorated many the effects of the drug use, especially preterm delivery rates. We have shown that increased prenatal visits, which roughly translates into participation with other clinic services is associated with increased abstinence and decreased relapse postpartum, though this could be a result of selection bias. Other authors have shown this with other drugs [51]. Quality prenatal care of at least 4 visits has been shown to significantly improve birth outcomes, decrease preterm delivery, and increase fetal birth weights [41]. Studies with cocaine-using pregnant women demonstrate that "comprehensive care" increases the likelihood of carrying to term, having fewer complications, being drug free at delivery, and having fewer exposed repeat pregnancies [52].

Our experience has shown us the importance of a comprehensive approach to treating women with addiction. It is important to address all components of the woman's life, including work and family: As one of our clients said, "I began using because I was working nights. My parents don't speak English and have many health problems, so I would have to get up after just and hour or two of sleep and take them to their doctors' appointments. I used ice [MA] to have energy to do that." Addressing nutrition is very important for example, as weight gain can be a powerful trigger for relapse, especially postpartum. One woman in our clinic stated that she was very unhappy with her weight postpartum which hadn't been a problem with her past pregnancies as she "just started using again and the weight came right off."

We believe a harm-reduction approach does work with these women, as we had high rates of engagement in services. As the clinic is in a house, many women stated they felt extremely comfortable there and would come when in crisis or just "to hang out." We had very high rates of abstinence during pregnancy, despite not mandating an abstinence-only approach. We believe this approach encourage honesty about their use. The women felt very comfortable discussing their use, and many times would disclose their relapses before a positive toxicology result was obtained.

Another success is the relatively low rates of post-partum depression, being fairly comparable to population-based rates of 14-21% [53,54], given the high incidence of antecedent depression in this population, and that a history of depression is one of the biggest risk factor in postpartum depression [53]. Having a safe, supportive environment postpartum was cited by many women as helpful to their general wellbeing.

The low rates of HIV and Hepatitis C point to the success of other harm reduction approaches; Hawaii was the first state in the nation to have a syringe-exchange program, which has been operating since 1990 and the majority of women in Hawaii smoke MA rather than inject.

The high rates of asthma are concerning, exceeding Hawaii's traditionally elevated rates of approximately 11% of the female population [55], and there are multiple explanations: higher asthma rates among Native Hawaiians; extremely high smoking rates in this population, and possibly the concomitant use of albuterol with MA to potentiate its effects. The high percentage of Native Hawaiians in this clinic population points to a large health disparity, which we have described before [30], and needs to be addressed with community-based research models.

The protective effect of residential treatment is seen within the drug use and custody domains. This result is to be expected as the residential treatment facility is the only one on the island of Oahu that allows women to bring a child into treatment with them. Women often chose this center in order to maintain custody. This type of program has demonstrated success in other areas and should be expanded.

The effect of a history of domestic violence on poor infant outcome is somewhat surprising, but not entirely unexpected, given high levels of psychosocial stress is associated with higher cortisol levels, and worse pregnancy outcomes [56]. Not surprising is the effect of maternal medical conditions on infant outcomes. Women should ideally enter pregnancy in optimal health, medically as well as mentally to insure the health of the infant.

While the majority of women were able to stop using drugs during pregnancy, they continued to smoke cigarettes. This is concerning given emerging data by us and others that smoking may be more harmful than MA use (at least on measurable pregnancy outcomes) [23] (Wright et al: *The placental and pregnancy effects of methamphetamines and smoking*, submitted) Chang, L. et al (personal communication, Hawaii Addictions Conference, March 19, 2010). While the numbers are too small to show any association with MA and congenital anomalies, it is reassuring that there were no large increases in these rates over baseline.

These women have high gravidity and parity, and a majority of these women have previously lost custody of at least one child. Most of the women in the "none" category were clients of a six-month residential treatment facility which permitted no sexual contact and thus thought themselves not to be at risk for pregnancy. Of the 78 women we have information on, only 17% became pregnant again within 18 months. Statewide, 21% of Medicaid women became pregnant within 15 months [57]. We showed that women who lost custody were 2.5 times more likely to not use reliable contraception and have repeat pregnancies. Helping women maintain custody seems to encourage women to chose reliable contraception. Our low repeat pregnancy rates is a great success.

There is room for improvement in the postpartum period, however, as 22% of the women were lost to follow-up. Of the women who did follow up, there was a 13% relapse rate to heavy usage at 6 months. Addition of pediatric care will help to keep women involved with the clinic, and allow long-term follow-up of the children and their outcomes.

## Conclusion

Methamphetamine use during pregnancy doesn't exist is isolation. It is often combined with a multitude of other adverse circumstances, including poverty, interpersonal violence, psychiatric comorbidity, polysubstance use, nutritional deficiencies, inadequate health care and stressful life experiences. A harm-reduction approach, which aims to ameliorate some of these difficulties for substance-using women without mandating abstinence, can work to engage women into prenatal care and normalize birth outcomes. This model provides an inexpensive and cost-effective approach (as the cost of one preterm infant can exceed the $200,000 annual cost of the program [56]) to affect community-wide change. Having a clinic available in the community seemed to improve screening rates for substance use disorders in the community physicians, as there was a resource available for referral of these often-marginalized women and their families. Directions for further study would be to compare a harm reduction approach to traditional treatment paradigm, e.g. adding cognitive behavioral therapy (CBT). Also a comparison study of MA-exposed pregnancies to non-MA exposed pregnancies will be possible with the addition of additional women in the non-MA group. Certainly the pediatric follow-up will add long-term longitudinal data on the effects of MA on the children and help guide interventions to ameliorate the damage.

## List of abbreviations

ACOG: American College of Obstetrics and Gynecology; AOR: adjusted odds ratio; CBT: cognitive behavioral therapy; CMV: cytomegalovirus; FAS: fetal alcohol syndrome; HIV: Human Immunodeficiency Virus; IDEAL: Infant, Development, Environmental, and Lifestyle; MA: methamphetamines; MRI: magnetic resonance imaging; NICU: newborn intensive care; PATH: Perinatal Addiction Treatment of Hawaii; PTSD: post traumatic stress disorder

## Consent

The University of Hawaii Committee on Human Studies reviewed the project and found it to be exempt from consent requirements in order to report clinical outcomes. A copy of the exemption is available for review by the Editor-in-Chief of the journal.

## Competing interests

The authors declare that they have no competing interests.

## Authors' contributions

TW obtained funding for clinic initiation, developed clinic design, designed study, drafted and submitted completed paper. RS assisted in clinic development, managed clinical operations, aided in data collection, and contributed to paper development. EF reviewed statistical analyses and developed outcome-scoring system. JS designed data collection form and completed the bulk of data collection. WFH assisted in the development of the clinic model, sat on the Path Clinic Board of Directors, provided psychiatric training and support. All authors read and approved the final manuscript.

## Author Information

TW is an Assistant Professor of Obstetrics, Gynecology and Women's Health and Clinical Assistant Professor of Psychiatry and Addiction Medicine at the University of Hawaii John A. Burns School of Medicine. RS was clinic manager and executive director of the Path Clinic. EF is Canada Research Chair Child Psychiatry, McGill University. WFH is Professor and Program Director in Addiction Psychiatry University of Hawaii John A. Burns School of Medicine.
